# A Study on Acetylglutamine Pharmacokinetics in Rat Blood and Brain Based on Liquid Chromatography-Tandem Mass Spectrometry and Microdialysis Technique

**DOI:** 10.3389/fphar.2020.00508

**Published:** 2020-04-30

**Authors:** Shouchao Xu, Chang Li, Huifen Zhou, Li Yu, Ling Deng, Jiazhen Zhu, Haitong Wan, Yu He

**Affiliations:** ^1^College of Pharmaceutical Sciences, Zhejiang Chinese Medical University, Hangzhou, China; ^2^College of Life Science, Zhejiang Chinese Medical University, Hangzhou, China

**Keywords:** liquid chromatography-tandem mass spectrometry, acetylglutamine, glutamic acid, γ-aminobutyric acid, microdialysis, blood-brain barrier

## Abstract

Acetylglutamine (NAG) is the derivative of glutamine, which is the richest free amino acid in the human body. In this work, a novel reliable method of the combination of liquid chromatography-tandem mass spectrometry (LC-MS/MS) and microdialysis (MD) technique for the evaluation of NAG and its metabolites γ-aminobutyric acid (GABA) and glutamic acid (Glu) in rat blood and brain was proposed. A Zorbax SB-C_18_ column (2.1 × 100 mm, 3.5 μM) was applied to separate the analytes. The mobile phase was acetonitrile-water (70:30, v/v) containing 5 mM ammonium acetate and the flow rate was 0.3 ml/min. Based on the multiple reaction monitoring (MRM) mode of positive ion, the precursors of product ions chosen for NAG, Glu, GABA, and N-carbamyl-L-glutamic (NCG, IS) were (m/z) 189.1→130.0, 148.0→84.1, 104→87.1, and 191.0→130.1, respectively. All the validation data, including precision, accuracy, inter-day repeatability, matrix effect, and stability, were within the acceptable ranges according to the reference of Bioanalytical Method Validation Guidance for Industry (2018). Rats with microdialysis probes inserted into jugular vein and hippocampus were administered the low (75 mg/kg, NAG-L), medium (150 mg/kg, NAG-M), and high (300 mg/kg, NAG-H) doses of NAG and 10 ml/kg Guhong injection (GHI) by tail vein, respectively. In the blood test, the C_max_ values of NAG-L group were markedly lower (*P* < 0.01) than those of NAG-M, NAG-H, and GHI groups, respectively. No differences were observed between NAG-M and GHI groups, while the C_max_ values in GHI group were significantly upgraded compared with NAG-H group. There were notable differences in the C_max_ values of NAG in brain dialysate after administration of NAG and GHI. The drug distribution coefficients of NAG, Glu, GABA in brain and blood at low, medium, high doses of NAG and GHI groups were 13.99, 27.43, 34.81, 31.37; 11.04, 59.07, 21.69, 2.69%; 212.88, 234.92, 157.59, and 102.65%, respectively. Our investigation demonstrates that NAG and its related metabolites in rat blood and brain can be simultaneously measured according to the above proposed method. Meanwhile, NAG has easy and dose-dependently access to the blood-brain barrier and exhibits a medium retention time in rat.

## Introduction

Accounting for about 60% of the total free amino acid in the human body, glutamine has the function to regulate the immune system and treat the gastrointestinal disturbances, clinically (Lopez-Pedrosa et al., 2007). Unfortunately, free glutamine is unstable in aqueous solution and easy to produce pyroglutamic acid which is harmful to human body. Acetylglutamine (N-acetyl-L-glutamine, NAG) is a derivative of glutamine and produced by the acetylation of glutamine (Hu, 2017). Regardless a harsh situation like sterilization or low pH, NAG shows better stability than glutamine and it can be made into freeze-dried powder to enhance its stability. The investigation of adding NAG to rat parenteral nutrition solution indicated that NAG was efficiently utilized to synthesize protein and hence might be served as a precursor for glutamine (Neuhauser and Bässler, 1986; Neuhauser-Berthold et al., 1988). The plasma level of glutamine doubled in dogs injected NAG through the vein. In addition, well tolerance and no obvious side effects were found in healthy volunteers and postoperative patients intravenously injected with NAG (Magnusson et al., 1989). NAG injection is mainly used as a brain function improving agent in the clinical to treat brain trauma, hepatic coma, hemiplegia, and sequelae of cerebral apoplexy (Liu and Wu, 2006; Yan et al., 2009). NAG is frequently combined with hydroxysafflor yellow A (HSYA, a main bioactive component of *Carthamus tinctorius* L.) to obtain better therapeutic effect (Ai et al., 2017). The major components of Chinese patent medicine like Guhong injection (GHI) are safflower and NAG, and the clinical value of GHI in ameliorating the neurological function of patients with cerebral infarction has been demonstrated. NAG can reach the hippocampus through the blood-brain barrier (BBB) to protect the delicate milieu of the brain because of its favorable lipophilicity. The medication instruction and related literature about NAG mention that γ-aminobutyric acid (GABA) and glutamic acid (Glu) are two of its metabolites of NAG (Yan et al., 2009; Yan and Lu, 2013; Liu et al., 2015). GABA can promote the activity of acetylcholine and improve brain function. While Glu, as an excitatory neurotransmitter, is closely related to the plasticity of neurons. It makes crucial effects on the growth of neurons and the formation of synapses, as well as the improvement of learning and memory (Lee and Kesner, 2002). In previous studies, the decomposition products of NAG were identified by mass spectrometry and nuclear magnetic resonance. NAG in the urine of rats or the intestinal infused solutions, intestinal mucosa, portal and peripheral blood of pigs was quantitated by HPLC (Hellmann et al., 1986; Bergana et al., 2000; Arnaud et al., 2004). However, the combination method of liquid chromatography-tandem mass spectrometric (LC-MS/MS) and microdialysis (MD) technique for analysis pharmacokinetic and NAG and its metabolites, Glu and GABA, in rat brain and blood, seems to be unknown within our scope.

On the other hand, MD is mature *in vivo* sampling technique and can continuously detect the concentration of endogenous and exogenous substance in peripheral extracellular fluid over time (Verbeeck, 2000). It is commonly used in the investigation of pharmacokinetics and unbound drugs in the hematological system, central nervous system (CNS), or other organs and tissues (Hammarlund-Udenaes et al., 1997; Muller et al., 1997; Rittenhouse et al., 1998; Verbeeck, 2000). Compared with traditional pharmacokinetic research methods, not only the consumption of animals notably reduced, but also the physiological function of the experimental animal would not be destroyed by using MD technique. Given the existence of the above advantages, a LC-MS/MS combined with MD sampling technology was established to characterize the pharmacokinetic features of NAG in rat brain and blood, meanwhile, the Glu and GABA which are the metabolites of NAG were also determined. The present study might provide a better understanding of the pharmacokinetics of NAG and related clinical medication.

## Materials and Methods

### Chemicals and Reagents

NAG was offered by Shanghai yuanye Bio-Technology Co., Ltd (Shanghai, China, purity ≥ 98%, batch number D05A6H3). Glu (purity ≥ 99%, batch number SLBC5771V) and GABA (purity ≥ 99%, batch number BCBH1414V) were purchased from SIGMA-ALDRICH (USA). NAG power injection was provided by Zhejiang Jinhua Conba Bio-Pharm Co., Ltd (Zhejiang, China, batch number DN1302002-4). GHI was the product of Tonghua Guhong Pharmaceutical Co., Ltd. with country medicine accurate character H22026582. *N*-carbamyl-*L*-glutamic (NCG, IS) was purchased from Hongxin Ruiyu Fine Chemical Co., Ltd. de Hubei (Hubei, China, purity > 98%, lot number 20160412). HPLC grade acetonitrile was obtained from Merck. A Milli-Q system manufactured by Millipore Corporation, Billerica, USA was applied for the preparation of ultra-pure grade water. Ringer's solution was prepared referring to previous literature (Kong et al., 2017). The fresh dialysate was gathered from drug free male Sprague-Dawley (SD) rats housed in our laboratory and maintained at −80°C until analysis. All other chemical reagents were analytical grade.

### Apparatus and Instruments

The material of brain and blood MD probes was polyethersulfone membrane and plastics, with 15-kDa cut-off and 0.6-mm outer diameter. The length of them was 4 mm and 10 mm, while the MBA was 6.14.4 and 7.8.10, respectively. Two kinds of MD probe were purchased from Microbiotech/se AB (Kista, Sweden) and used throughout the studies. Micro-infusion pump was purchased from BAS (USA, IN47906).

### Animals

Adult male SD rats (weighting 250–300g) were provided by the Animal Center of Zhejiang Chinese Medical University (Laboratory Animal Certificate: scxk 2014-0001). The rats were raised in specific pathogen free (SPF) animal room with suitable temperature and humidity. The animals were supplied free food and water and demanded to acclimatize to the light-dark cycle of 12 h. The Guidelines for the Care and Use of Laboratory Animals, prepared by the National Academy of Sciences and published by the National Institutes of Health, were followed to care for all animals. The animal procedures, approved by the institutional animal care and use committee of Zhejiang Chinese Medical University, were applied to all animals. All efforts were made to reduce the number of animals and minimize pain, suffering, and distress.

### Stock Solutions

NAG, Glu, and GABA were respectively dissolved in Ringer's solution to prepare the stock solution with the concentration of 1 mg/ml. Diluting the Ringer's solution, the stock solution was further prepared as a series working standard solutions of NAG, Glu, and GABA whose concentration ranges were 1–10,000, 10–50,000, and 10–50,000 ng/ml, respectively. The IS solution at a concentration of 0.85 μg/ml was prepared by dissolving NAG in Ringer's solution. The temperature of −4 °C was maintained for all solutions until analysis. The fresh calibration standards with the concentrations of 1, 10, 50, 250, 500, 1,000, 5,000, 10,000 ng/ml were prepared by diluting the stock solution of NAG on each experimental day. The calibration standards of Glu, GABA were freshly prepared at 10, 50, 250, 500, 1,000, 5,000, 10,000, 50,000 ng/ml following the same procedure, respectively. The quality control (QC) samples of the mixture of three components (NAG, Glu, and GABA) were prepared daily in the same manner as calibration standard, and their concentration levels were 50 ng/ml (low dose), 500 ng/ml (middle dose), 5,000 ng/ml (high dose), respectively.

### Experimental Design

All rats were randomly classified into five groups (six rats per group): blank group, NAG-L group (NAG, 75 mg/kg), NAG-M group (NAG, 150 mg/kg), NAG-H group (NAG, 300 mg/kg), and GHI group (10 ml/kg). All these doses of NAG were dissolved in 1 ml saline respectively. Drugs and saline were administered by tail vein. Sham group was injected with the same volume of saline.

### LC-MS/MS Analysis

#### HPLC Conditions

The HPLC chromatographic separation was achieved through a Shimadzu apparatus (Kyoto, Japan) with system controller (CBM-20A), pump (LC-30AD), autoinjector (SIL-30AC), online degasser (DGU-20A), and column heater (CTO-20A). An Agilent Zorbax SB-C_18_ column with the diameter of 2.1 × 100 mm (3.5 μM, Agilent, USA) was applied to separate the analytes. The temperature of column was 40°C. Analytes were equivalently eluted with 30:70 (v/v) acetonitrile-water containing 5 mM ammonium acetate (pH 4) and the flow rate was 0.3 ml/min. The analysis time of all analytes was 1.8 min, and the injection volume was 10 μl.

#### MS Conditions

Analytes in rat blood and brain dialysate were detected using an AB SCIEX QTRAP 4500 mass spectrometer with electrospray ionization (ESI) source (AB Sciex, USA). Data was acquired by Multi Quant 2.1 software. Positive ion multiple reactions monitoring (MRM) mode with ion spray voltage of 5,500 eV, heated capillary temperature of 500°C, sheath gas (nitrogen) of 50 psi, and auxiliary gas (nitrogen) of 50 psi was used to detect the analytes. The parent ions to the product ions chosen for NAG, Glu, GABA, and IS quantification were (m/z) 189.1→130.0, 148.0→84.1, 104→87.1, and 191.0→130.1, respectively.

### Method Validation

#### Specificity

Six blank dialysate from different rats, blank dialysate spiked with the analytes and IS, and dialysate from a random rat with administration of NAG were analyzed to verify the specificity of the established method. This study was made to ensure there were no potential components to disturb the analysis of NAG, Glu, GABA, and IS.

#### Linearity and LLOQ

In order to detect the method linearity, three replicates with eight different concentration levels of NAG, GABA, Glu were analyzed over 3 consecutive days. According to the weighted least square linear regression, the calibration curves were plotted as peak area ratios (*y*) of the analyte to IS versus the nominal concentrations of the analyte (*x*). LLOQ was the lowest concentration of the calibration curve with S/N greater than 10.

#### Inter-Day and Intra-Day Precision and Accuracy

Six replicates of the QC samples at each concentration on the same day were determined to evaluate the intra-day precision and accuracy. The same procedure over 5 consecutive days was employed to evaluate the inter-day precision and accuracy. The accuracy and precision were evaluated by the relative error (RE%) and relative standard deviation (RSD %), respectively, and their values should be within the range of ±15% (U.S. Department of Health and Human Services, Food and Drug Administration, Center for Drug Evaluation and Research, Center for Veterinary Medicine, 2018).

#### Matrix Effect

Matrix Effect (ME) was defined as the ratio between the peak area of the analytes in blank samples and that in neat solution. The peak area (B) was obtained by determining the six different blank dialysate spiked with the mixed QC samples of the analytes concentrations at 50, 500, 5,000 ng/ml. The dialysate was replaced by pure water, by the same procedure, the peak area (C) was achieved by adding the same concentrations of QC samples. The ME of the IS was also assessed at the working concentration (8.5 μg/ml) by the same procedure. ME was calculated by B/C × 100%. The ratio should be within the range of 85~115% (U.S. Department of Health and Human Services, Food and Drug Administration, Center for Drug Evaluation and Research, Center for Veterinary Medicine, 2018).

#### Stability

The stability of NAG, Glu, GABA was evaluated by analyzing six replicates at three different concentrations QC samples containing NAG, Glu, GABA under different storage conditions. The stability of short-term and long-term was obtained by elucidating the QC samples at 4°C for 12 h, and at −80°C for 7days, respectively. The QC samples were consecutively frozen and thawed three times to assess the freeze/thaw stability.

### Experimental Animals and Samples Collection

Urethane (20%, 1 g/kg) was used to anesthetize the SD rats by intraperitoneal injection. With the body temperature kept at 37°C by heating light, the rats were put in a stereotaxic frame and their heads were fixed. The dura mater on skull was cut with a scalpel and the sagittal suture was exposed. Then the stereotaxic coordinate was used to drill a small hole on the skull. The intra-cerebral guided intubation was embedded into the hippocampus area (coordinates: AP, anteroposterior, −4.8 mm; ML, mediolateral, +/−5 mm; DV, dorsoventral, −3 mm) according to the Paxinos and Watson atlas. Finally, the tube was fixed by tooth powder and dehydration. All surgical procedures were performed with aseptic technique. The standard histological procedure was performed to verify the position of the probes in the hippocampus area at the end of the studies.

The rats recovered for 3 days, and then 20% urethane (1.2 g/kg) was used to anesthetize the rats. Brain and blood MD probes were positioned in the intra-cerebral guide cannula and the right jugular vein toward the right atrium, respectively. Following the implantation of MD probes, Ringer's solution was perfused into the probes using the micro-infusion pump at 1.5 μl/min for 60 min to balance the system and achieve blank samples. After the blank duration, NAG (75, 150, 300 mg/kg) or GHI (10 ml/kg) was administered by tail vein. The MD samples were gathered every 20 min for 6 h and maintained at −80°C until analysis. After the end of the experiment, the probes were taken out from the brain and the rats were euthanized. The brain was extracted to confirm the probe placement.

### *In Vivo* Relative Recovery

MD probe *in vitro* recovery (RR *_in vitro_*), *in vitro* loss (RL *_in vitro_*), and *in vivo* loss (RL *_in vivo_*) were detected for the calculation of probe *in vivo* relative recovery (RR *_in vivo_*).

RR *_in vitro_* of NAG, Glu, and GABA were carried out by placing the blood and brain probes in 0.2, 0.4, 0.6, 0.8, and 1 μg/ml of the NAG, Glu, and GABA mixture quiescent solutions respectively which were magnetically stirred at 350 rpm at room temperature and perfusing Ringer's solution at a flow rate of 1.5 μ;/min. After equilibration for 1 h, the dialysate of each concentration was gathered five times and measured to obtain RR *_in vitro_*. The percent RR *_in vitro_* versus concentration was calculated in the dialysate (C_d_) and perfusate medium (C_p_).

Ringer's solution containing the NAG, Glu, and GABA mixture (10 μg/ml, respectively) passed through the blood and brain microdialysis probes, which were placed in a beaker containing Ringer's solution, and the dialysate was gathered and analyzed by established LC-MS/MS method to obtain RL *_in vitro_*.

By the similar way, probe RL *_in vivo_* of brain and blood dialysate was obtained using the retrodialysis calibration technique. MD probes were positioned into the striatum and the right jugular vein under anesthesia. After the implantation of MD probes, Ringer's solution was perfused through the probes and the flow rate was 1.5 μl/min. The system was equilibrated for 1 h before sample collection. Subsequently, Ringer's solution containing 1 μg/ml NAG was perfused into the probes at a flow rate of 1.5 μl/min. Microdialysate was gathered at every 30 min. After 6 h of probes implantation, the perfusate and dialysate concentrations of NAG were detected by LC-MS/MS. The probe relative recovery (RR) and relative loss (RL) were calculated according to Eq (1). The probe RR *_in vivo_* was defined by the following Eq (2). The concentrations of NAG, Glu and GABA detected in the microdialysate (C_m_) were corrected to concentrations (C_f_) in the blood and brain as the Eq (3).

(1) $$\text{RR}=(C_{d}/C_{p})\times 100\%;\;\text{RL}=(C_{p}-C_{d})/C_{p}\times 100\%$$

(2) $${\text{RR}}_{in\;vivo}/{\text{RL}}_{in\;vivo}={\text{RR}}_{in\;vitro}/{\text{RL}}_{in\;vitr\text{o}}$$

(3) $${\text{C}}_{\text{f}}={\text{C}}_{\text{m}}/{\text{RR}}_{in\;vivo}$$

### Statistical Analysis

Mean ± standard deviation (S.D.) was used to express the value of each group. SPSS 17.0 statistical analytical software (SPSS Inc) was used to analyze the data. A one-way ANOVA was used to assess the experimental data. *P* value of less than 0.05 was supposed to be statistically significant. The pharmacokinetic parameters of NAG were calculated by PK Slover (version 2.0; China Pharmaceutical University) using the non-compartmental analysis.

## Results

### Method Validation

**Figure 1:**
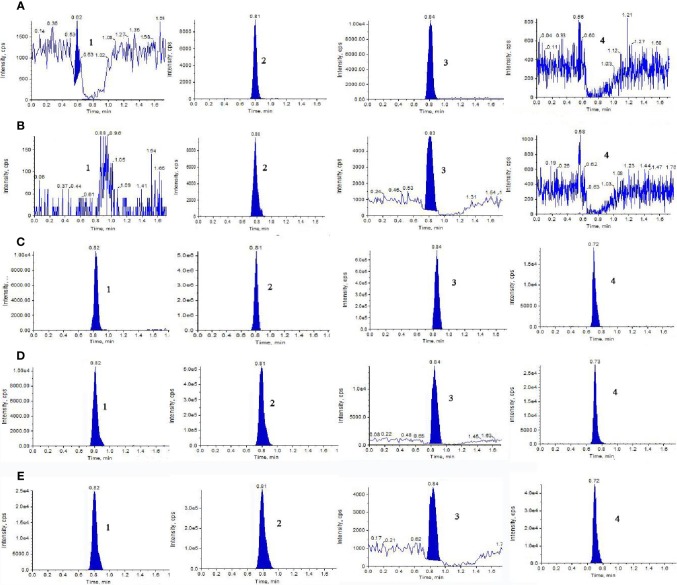
Representative extract ion MRM chromatograms of (1) NAG, (2) Glu, (3) GABA, and (4) IS in blood and brain microdialysis solution: **(A)** blank blood dialysate; **(B)** blank brain dialysate; **(C)** QC solution; **(D)** blood dialysate sample; **(E)** brain dialysate sample obtained at a flow rate of 0.3 ml/min with the mobile phase composed of acetonitrile-water (70:30, v/v) containing 5mM ammonium acetate.

Representative extract ion MRM chromatograms of blank dialysate, blank dialysate spiked with 500 ng/ml stock solutions of NAG, Glu, and GABA, and the microdialysate gathered from a random rat which was administrated NAG or GHI were shown in **Figure 1**. The retention times of the analytes and IS were 0.82, 0.81, 0.84, and 0.73 min, respectively. The results of specificity indicated that there were no significant endogenous interferences at the retention times of NAG, Glu, GABA, and IS peaks.

Regression equations, linear ranges, and correlation coefficients (*r*) of the analytes were listed in **Table 1**. All calibration curves reveal good linearity and the *r* values were >0.99.

**Table 1:** Linearity for assay of NAG, Glu, and GABA.

Analyte	Linear range (ng/ml)	Regression equation	Correlation coefficient	Weight
NAG	1–10,000	y = 0.0001×−0.0014	0.9990	1/χ2
Glu	10–50,000	y = 0.0075×+3.1115	0.9991	1/χ2
GABA	10–50,000	y = 0.0083×+0.0056	0.9999	1/χ2

With the aim to determine the intra-day and inter-day precision (RSD%) and accuracy (RE%), six dialysate QC samples at the concentration of 50, 500, 5,000 ng/ml were analyzed within 1 day and 5 consecutive days, respectively. As shown in **Table 2**, the RSD% of intra- and inter-day precision was between 1.05 and 7.31%, while RE% of accuracy ranged from −3.23 to 5.91%, which were all acceptable.

**Table 2:** Intra-day and inter-day precision (RSD %) and accuracy (RE %) of the LC-MS/MS method for determination of NAG, Glu, and GABA in QC solutions (n = 6).

Analyte	Nominal Concentration (ng/ml)	Intra-day			Inter-day		
		Observed concentration (ng/ml)	Precision (RSD %)	Accuracy (RE %)	Observed concentration (ng/ml)	Precision (RSD %)	Accuracy (RE %)
NAG	50	50.81 ± 1.56	3.07	1.63	50.67 ± 1.65	3.27	1.35
	500	492.36 ± 14.91	3.03	−1.53	499.79 ± 9.72	1.95	−0.04
	5,000	5065.94 ± 71.49	1.41	1.32	5,036.24 ± 117.44	2.33	0.72
Glu	50	51.03 ± 1.60	3.15	2.06	50.76 ± 2.18	4.29	1.53
	500	483.84 ± 5.06	1.05	−3.23	507.59 ± 14.54	2.87	1.52
	5,000	5295.54 ± 282.39	5.33	5.91	4,986.62 ± 143.07	2.87	0.27
GABA	50	50.56 ± 1.22	2.14	1.12	50.69 ± 1.98	3.90	1.39
	500	527.92 ± 38.58	7.31	5.58	504.00 ± 13.53	2.69	0.80
	5,000	5175.83 ± 267.90	5.18	3.52	5,001.52 ± 200.45	4.00	0.03

The results of stability were summarized in **Table 3**. A maximum deviation of QC solutions was 9.57%, which showed that NAG, Glu, and GABA were stable under all conditions employed in this study.

**Table 3:** Stability of NAG, Glu, and GABA under different storage conditions (n = 6).

Storage condition	Nominal concentration (ng/ml)	NAG		Glu		GABA	
		Calculated concentration (ng/ml) (mean ± SD)	RSD (%)	Calculated concentration (ng/ml) (mean ± SD)	RSD (%)	Calculated concentration (ng/ml) (mean ± SD)	RSD (%)
The freeze-thaw cycles at −20°C	50	52.64 ± 3.15	5.99	50.84 ± 4.86	9.57	52.51 ± 3.06	5.83
	500	506.44 ± 29.72	5.87	495.74 ± 17.47	3.52	506.83 ± 31.98	6.31
	5,000	4,995.12 ± 219.92	4.40	5,037.66 ± 342.16	6.79	5,001.39 ± 426.47	8.53
Storage for 7 days at −80°C	50	51.65 ± 2.86	5.00	50.44 ± 2.96	5.87	49.63 ± 2.02	4.07
	500	501.26 ± 8.22	1.64	520.63 ± 14.38	2.76	503.37 ± 11.71	2.32
	5,000	5,060.85 ± 187.06	3.70	5,118.27 ± 238.00	4.65	5,001.62 ± 289.48	5.79
Storage for 24 h at −4°C	50	52.55 ± 2.49	4.75	52.59 ± 2.51	4.78	51.17 ± 1.97	3.84
	500	503.42 ± 13.20	2.62	513.92 ± 13.07	2.54	516.67 ± 16.25	3.15
	5,000	5,089.05 ± 138.95	2.73	4,960.40 ± 111.99	2.26	5,039.71 ± 240.78	4.78

The post-extraction spike method was performed to detect the ME. The results were shown in **Table 4** indicated that there was no endogenous substance to affect the reproducibility and the accuracy of the analysis in the rat brain or blood dialysate.

**Table 4:** Matrix effect of NAG, Glu, GABA, and the IS in rat brain and blood dialysate (n = 6).

Analytes	Spiked/ng/ml	Brain		Blood	
		Mean ± S.D. (%)	RSD (%)	Mean ± S.D. (%)	RSD (%)
NAG	50	102.06 ± 3.26	3.20	105.22 ± 4.99	4.75
	500	100.96 ± 5.56	5.76	97.71 ± 5.16	5.28
	5,000	101.17 ± 4.92	4.86	102.21 ± 1.97	1.93
Glu	50	106.56 ± 1.87	1.76	107.05 ± 3.28	3.06
	500	110.15 ± 1.79	1.62	105.19 ± 3.70	3.52
	5,000	102.85 ± 3.86	3.75	103.38 ± 2.31	2.24
GABA	50	103.12 ± 3.77	3.66	106.34 ± 2.36	2.22
	500	104.31 ± 3.35	3.22	109.82 ± 2.01	1.83
	5,000	98.47 ± 3.28	3.33	101.87 ± 3.84	3.76

The detected results of RR%, RL% indicated that the increased flow rate from 1 to 4 μl/min lead to a decrease in RR% and RL% (**Tables 5** and **6**). Consequently, the flow rate was optimized and the optimal flow rate was 1.5 μl/min for further *in vivo* study of NAG, Glu, GABA. *In vitro* average recovery of dialysis and retrodialysis was displayed in **Table 7**. The data indicated that there was no relationship between relative recovery and loss and concentration. However, the data was inversely related to the perfusion flow rate.

**Table 5:** The blood probes *in vitro* relative recovery (RR) and relative lose (RL) of NAG, GABA, and Glu in different flow rates.

Flow rate (μl/min)	NAG		Glu		GABA	
	RR (%)	RL (%)	RR (%)	RL (%)	RR (%)	RL (%)
1	21.79 ± 1.69	22.19 ± 2.35	26.24 ± 1.16	24.78 ± 1.19	46.77 ± 1.72	47.93 ± 2.32
1.5	12.72 ± 1.50	13.99 ± 2.07	20.91 ± 2.18	20.23 ± 1.64	34.27 ± 2.74	35.22 ± 3.22
2	8.55 ± 1.60	8.30 ± 1.50	17.98 ± 0.97	18.75 ± 3.30	31.84 ± 3.12	30.66 ± 4.29
3	4.91 ± 0.97	5.30 ± 1.62	13.59 ± 0.72	14.07 ± 1.21	23.48 ± 1.20	23.62 ± 3.19
4	2.86 ± 0.29	3.06 ± 0.94	11.26 ± 0.70	11.75 ± 1.32	16.43 ± 1.52	14.51 ± 1.63

**Table 6:** The brain probes *in vitro* relative recovery (RR) and relative lose (RL) of NAG, GABA, and Glu in different flow rates.

Flow rate (μl/min)	NAG		Glu		GABA	
	RR (%)	RL (%)	RR (%)	RL (%)	RR (%)	RL (%)
1	52.44 ± 1.62	52.12 ± 5.65	53.45 ± 3.91	56.20 ± 2.12	61.84 ± 3.02	62.85 ± 2.31
1.5	39.16 ± 1.78	38.90 ± 4.61	42.39 ± 2.73	44.47 ± 3.30	48.49 ± 3.10	48.66 ± 2.32
2	22.24 ± 2.53	22.07 ± 3.89	35.16 ± 3.53	36.50 ± 2.42	36.88 ± 3.01	39.36 ± 3.63
3	16.42 ± 3.03	16.31 ± 1.39	26.90 ± 2.50	27.22 ± 4.91	26.61 ± 1.87	24.43 ± 3.87
4	9.49 ± 1.79	8.06 ± 2.45	22.67 ± 2.49	20.11 ± 4.41	22.65 ± 2.19	18.72 ± 1.81

**Table 7:** The blood and brain probes *in vitro* relative recovery (RR) and relative lose (RL) of NAG, GABA, and Glu in different concentrations.

Concentration (μg/ml)	Blood probe			Brain probe		
	NAG	Glu	GABA	NAG	Glu	GABA
0.4	37.69 ± 2.20	44.40 ± 4.33	42.17 ± 1.34	12.47 ± 2.66	21.49 ± 2.78	34.76 ± 1.81
0.8	38.37 ± 3.93	41.44 ± 1.92	48.07 ± 1.43	11.39 ± 1.27	21.16 ± 2.38	35.31 ± 3.38
1.2	41.09 ± 2.15	42.28 ± 2.37	49.00 ± 1.67	12.47 ± 1.24	17.05 ± 2.46	31.47 ± 2.70
1.6	36.65 ± 3.62	43.31 ± 1.34	49.38 ± 4.10	12.88 ± 0.75	22.30 ± 0.89	35.21 ± 1.80
2	39.13 ± 1.24	40.87 ± 3.50	49.15 ± 2.21	11.25 ± 2.33	21.31 ± 3.05	35.07 ± 2.54
Average	38.59 ± 1.67	42.46 ± 1.42	47.55 ± 3.05	12.09 ± 0.73	20.66 ± 2.07	34.36 ± 1.63

**Figure 2:**
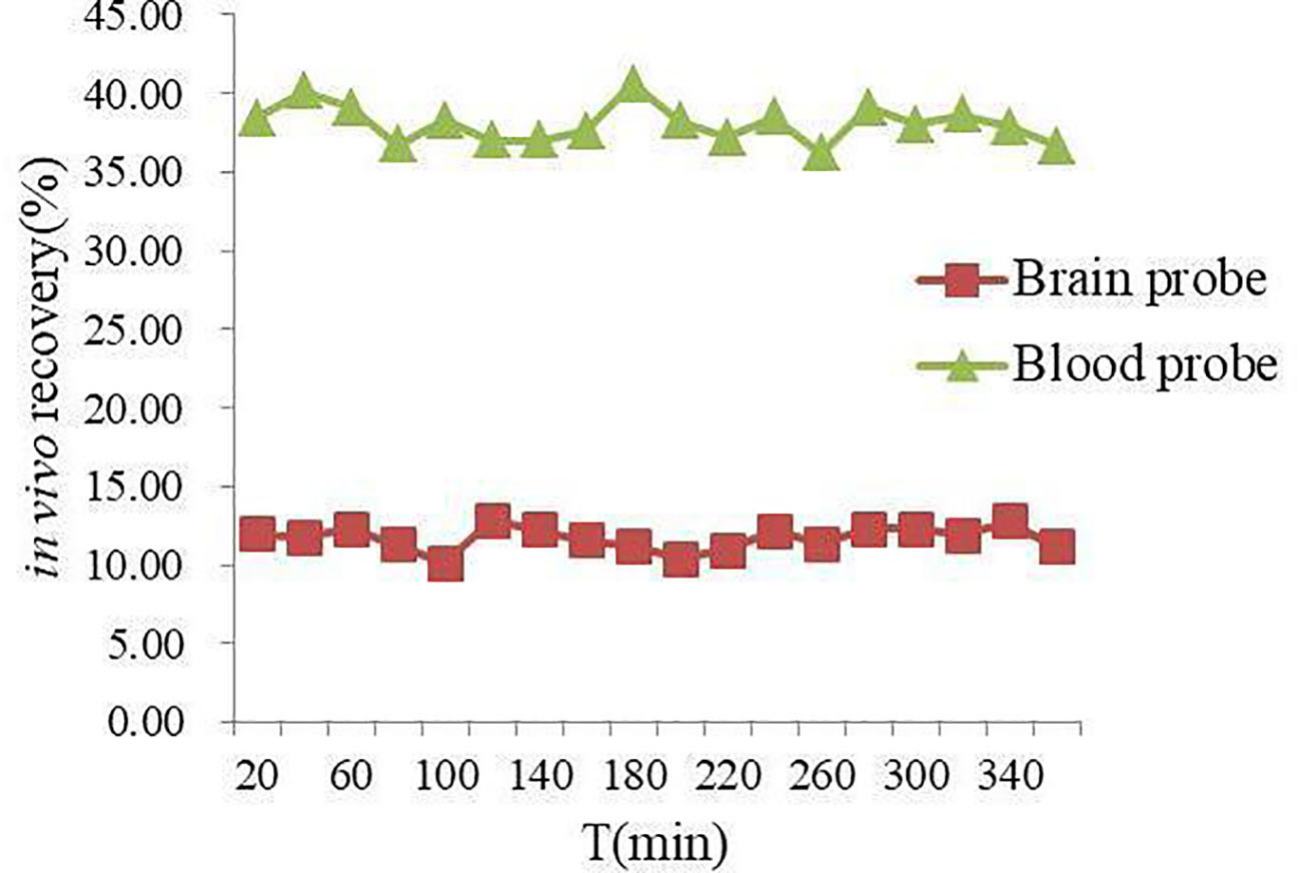
*In vivo* recovery (%) of NAG in rat blood and brain probes.

*In vivo* NAG recovery of MD probe in rat blood and brain at different time points was illustrated in **Figure 2**. The mean *in vivo* recovery of NAG in rat blood and brain was (38.07 ± 3.73) % and (11.67 ± 1.95) %, respectively. It could be seen clearly from the values of the *in vivo* recovery that NAG was stable within 6 h, prompting that microdialysate samples should be gathered after equilibrium of probe implantation.

### Pharmacokinetics of NAG, Glu, and GABA in Rat Brain and Blood

**Figure 3:**
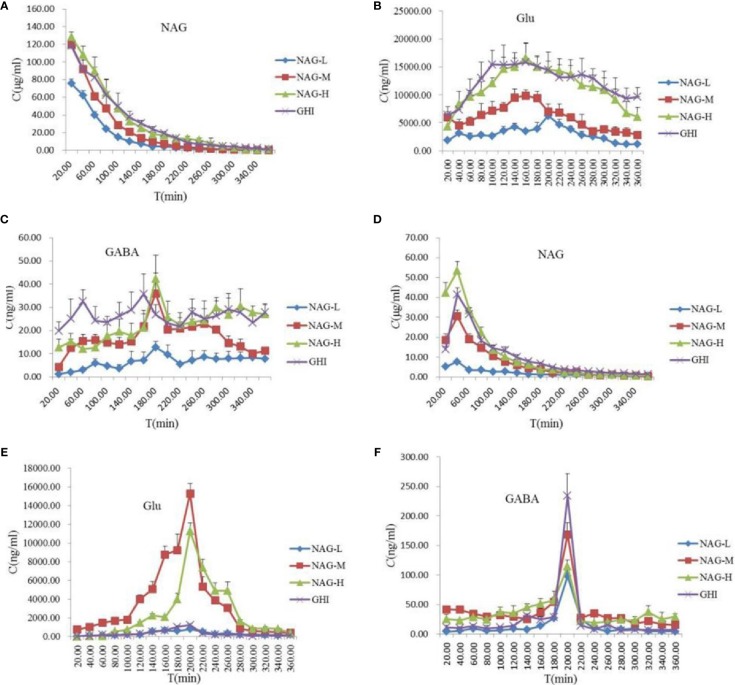
Concentration-time profiles of protein-unbound NAG **(A)** and Glu **(B)**, GABA **(C)** in rat blood, and NAG **(D)**, Glu **(E)**, GABA **(F)** in rat brain dialysates after tail vein administration of 75 mg/kg NAG (NAG-L), 150 mg/kg NAG (NAG-M), 300 mg/kg NAG (NAG-H), and GHI (10 ml/kg). Data are expressed as mean ± S.D. (n = 6).

NAG, Glu, and GABA in rat brain and blood samples were quantitatively analyzed and the concentration-time profiles were shown in **Figure 3**. The established LC-MS/MS method has been successfully validated and applied to the rat brain and blood pharmacokinetic study of the analytes. Brain and blood pharmacokinetic parameters including the area under the MD sample concentration-time curve (AUC), average dwell time (MRT), half-life (T_1/2_), peak time (T_max_), and maximum concentration (C_max_) were calculated. All the tested NAG, Glu, and GABA were detectable in blood and brain up to 360 min post dosing.

NAG with the small molecular weight and the low protein binding rate might make it has good BBB permeability. In the rat blood dialysate (**Figure 3A**, **Table 8**), the C_max_, AUC_0-t_, AUC_0-∞_ values of NAG in the NAG-L group were markedly lower than those of the NAG-M, NAG-H, and GHI groups (all *P* < 0.01). There were no differences between NAG-M and GHI groups, while, the C_max_ in GHI group was notably downgraded compared with NAG-H group (*P* < 0.05). The AUC_0-t_ and AUC_0-∞_ values of NAG-M were obviously lower compared to NAG-H and GHI groups. No differences were found between NAG-H and GHI groups in AUC_0-t_ and AUC_0-∞_ values. The values of T_1/2_ in the NAG-H group conspicuously decreased, compared with those in NAG-L and GHI groups. In comparison with NAG-L group, a remarkable increase in the values of MRT was observed in NAG-H and GHI groups (all *P* < 0.01). Significant differences in pharmacokinetic parameters of NAG in brain dialysate after administration of NAG (75, 150, 300 mg/kg) and GHI (10 ml/kg) including C_max_, MRT, AUC_0-t_, AUC_0-∞_ (**Figure 3D**, **Table 8**) were observed. Compared with NAG-L group, the values of T_1/2_ in NAG-M, NAG-H, and GHI groups were markedly decreased (all *P*<0.01). In comparison with NAG-H group, the T_1/2_ values of GHI group significantly increased (P < 0.05).

**Table 8:** Pharmacokinetic parameters of NAG in blood and brain dialysate after administration of NAG (75, 150, 300 mg/kg) and GHI (10 ml/kg) (mean ± S.D., n = 6).

Parameters	Unit	NAG-L	NAG-M	NAG-H	GHI
**blood**					
C_max_	μg/ml	74.35 ± 4.40	118.61 ± 6.67	128.25 ± 6.24	118.37 ± 6.50
T_max_	min	20	20	20	20
AUC_0-t_	min μg/ml	5,974.85 ± 308.14	9,912.29 ± 279.02	13,231.38 ± 879.57	12,513.72 ± 1076.02
AUC_0-∞_	min μg/ml	5,995.84 ± 307.47	9,930.36 ± 276.77	13,296.37 ± 912.14	12,585.28 ± 1088.65
T_1/2_	min	44.15 ± 3.19	38.30 ± 6.89	30.23 ± 11.86	45.33 ± 13.44
MRT	min	56.06 ± 1.73	56.65 ± 2.42	74.24 ± 4.03	77.43 ± 3.75
**brain**					
C_max_	μg/ml	7.76 ± 0.50	30.57 ± 3.33	53.44 ± 4.71	41.48 ± 3.34
T_max_	min	40	40	40	40
AUC_0-t_	min μg/ml	835.63 ± 22.90	2,719.18 ± 126.95	4,584.42 ± 233.23	3,772.27 ± 235.01
AUC_0-∞_	min μg/ml	978.10 ± 58.06	2,754.50 ± 122.00	4,644.40 ± 233.96	4,016.83 ± 420.52
T_1/2_	Min	120.35 ± 17.88	57.80 ± 3.35	57.39 ± 6.03	74.81 ± 14.84
MRT	Min	170.61 ± 24.99	86.37 ± 1.81	73.60 ± 3.35	73.60 ± 3.35
AUC_brain_/A	—	13.99 ± 0.6	27.43 ± 1.47	34.81 ± 3.65	34.81 ± 3.65
AUC_blood_/%					

The content and pharmacokinetic parameters of Glu after administrated with NAG and GHI were shown in **Figures 3B, E** and **Table 9**. In the blood dialysate, the values of C_max_, AUC_0-t_, AUC_0-∞_, and T_1/2_ in NAG-L group were significantly lower than those in NAG-M and NAG-H groups. The values of MRT in NAG-M group were slightly increased compared with NAG-L, while the MRT values in NGA-H group were significantly higher than those of NAG-L group. In comparison to the NAG-M group, a marked rise in the values of C_max_, AUC_0-t_, AUC_0-∞_, T_1/2_, and MRT was observed in the GHI group. No significant differences existed between NAG-H and GHI groups in the C_max_ and AUC_0-t_ values. Remarkably, the AUC_0-∞_, T_1/2_, and MRT values in GHI group were higher than those in NAG-H group (*P*<0.001, respectively). From the brain dialysate, the values of C_max_, AUC_0-t_, and AUC_0-∞_ in NAG-L group were notably decreased compared to the NAG-M and NAG-H groups. There were no significant differences among NAG-L, NAG-M, and NAG-H groups with the values of MRT. Compared with NAG-L group, the C_max_ values in GHI group were significantly increased, the values of AUC_0-t_, AUC_0-∞_, T_1/2_, and MRT in GHI group were similar to NAG-L group.

**Table 9:** Pharmacokinetic parameters of Glu in blood and brain dialysate after administration of NAG (75, 150, 300 mg/kg) and GHI (10 ml/kg) (mean ± S.D., n = 6).

Parameters	Unit	NAG-L	NAG-M	NAG-H	GHI
**blood**					
C_max_	μg/ml	5,566.33 ± 1,102.14	9,999.94 ± 975.78	17,499.67 ± 1,605.09	17,999.53 ± 1,541.07
T_max_	min	200 ± 20	140 ± 22.95	192 ± 33.47	130 ± 27.57
AUC_0-t_	min μg/ml	1,077.47 ± 71.02	2,194.84 ± 122.12	4,078.53 ± 246.03	4,411.36 ± 533.31
AUC_0-∞_	min μg/ml	1,188.00 ± 98.42	2,654.84 ± 213.90	4,934.92 ± 395.20	7,578.10 ± 735.28
T_1/2_	min	64.04 ± 1.82	108.84 ± 5.21	86.50 ± 8.89	279.40 ± 17.83
MRT	min	198.27 ± 9.67	223.43 ± 12.27	237.12 ± 16.18	529.41 ± 26.11
**brain**					
C_max_	μg/ml	940.73 ± 95.22	15,416.29 ± 1,170.58	11,275.20 ± 895.12	1,206.82 ± 91.39
T_max_	min	200	200	200	200
AUC_0-t_	min μg/ml	118.76 ± 6.33	1,296.60 ± 51.96	884.69 ± 27.03	118.74 ± 4.11
AUC_0-∞_	min μg/ml	141.59 ± 8.36	1,314.58 ± 48.45	902.94 ± 28.41	132.18 ± 6.89
T_1/2_	Min	67.30 ± 4.13	30.39 ± 1.17	32.99 ± 1.02	60.52 ± 6.15
MRT	Min	234.35 ± 9.60	182.01 ± 1.44	215.90 ± 2.18	214.54 ± 14.69
AUC_brain_/A	—	11.04 ± 0.50	59.07 ± 2.78	21.69 ± 0.99	2.69 ± 0.34
AUC_blood_/%					

As illustrated in **Figure 3C** and **Table 10**, the values of C_max_ in blood dialysate of NAG-H group were markedly higher than those of other administrated groups. Compared with NAG (75, 150, 300 mg/kg) groups, the T_1/2_ and MRT values in GHI group were obviously upgraded. The content and pharmacokinetic parameters of the GABA in brain dialysate were shown in **Figure 3F** and **Table 10**. The C_max_ values in GHI group were significantly increased in comparison with NAG groups. Inversely, the values of MRT in GHI were dramatically lower than those in NAG groups. In comparison with GHI group, the AUC_0-t_ and T_1/2_ values in NAG-L group were significantly lower, while in NAG-M and NAG-H groups, the values of AUC_0-t_ and

**Table 10:** Pharmacokinetic parameters of GABA in blood and brain dialysate after administration of NAG (75, 150, 300 mg/kg) and GHI (10 ml/kg) (mean ± S.D., n = 6).

Parameters	Unit	NAG-L	NAG-M	NAG-H	GHI
**blood**					
*C*_max_	μg/ml	14.15 ± 1.86	35.48 ± 3.26	46.25 ± 7.57	37.25 ± 3.04
*T*_max_	min	186.67 ± 11.55	173.33 ± 11.54	208 ± 62.61	153.33 ± 11.55
AUC_0-t_	min μg/ml	2,439.22 ± 297.17	5,984.99 ± 269.87	8,211.13 ± 996.86	9,257.38 ± 713.92
AUC_0-∞_	min μg/ml	4,603.47 ± 381.58	7,362.57 ± 204.04	11,932.80 ± 369.29	22,111.95 ± 2,652.87
T_1/2_	min	155.05 ± 5.79	84.21 ± 7.21	143.69 ± 12.41	311.12 ± 2.68
MRT	min	232.16 ± 9.95	245.38 ± 7.78	285.15 ± 38.46	541.11 ± 6.53
**brain**					
*C*_max_	μg/ml	98.39 ± 10.74	174.94 ± 16.70	114.63 ± 11.03	244.89 ± 13.17
*T*_max_	min	200	200	200	200
AUC_0-t_	min μg/ml	5,192.49 ± 475.31	14,060.00 ± 407.05	12,939.70 ± 765.62	9,502.58 ± 823.02
AUC_0-∞_	min μg/ml	5,533.03 ± 506.78	16,025.80 ± 1518.08	21,640.60 ± 1785.04	10,132.60 ± 879.29
T_1/2_	Min	46.46 ± 5.58	83.82 ± 6.56	164.25 ± 14.99	65.93 ± 9.02
MRT	Min	205.52 ± 16.04	207.42 ± 25.71	328.15 ± 22.35	198.30 ± 3.05
AUC_brain_/A	—	212.88 ± 46.80	234.92 ± 9.21	157.59 ± 26.89	102.65 ± 10.56
AUC_blood_/%					

T_1/2_ were notably higher. The MRT values in NAG-H group were markedly increased compared with GHI group (*P*<0.01). There were no differences between NAG-H and GHI groups in the AUC_brain_/AUC_blood_ values of NAG, and compared with NAG groups, the AUC_brain_/AUC_blood_ ratio of Glu in GHI was dramatically decreased.

## Discussion

Due to the high plasma protein binding rate of NAG, the common detection methods are unable to accurately monitor its content changes in the body. However, LC-MS/MS which employed liquid chromatography as a separation system and mass spectrometry as a detection system is quick, simple, and sensitive to determine the NAG, Glu, and GABA in rat blood or brain microdialysate. The established LC–MS/MS method was validated and good specificity, linearity, precision and accuracy, stability of NAG, Glu, and GABA were observed. Aceglutamide has two configurational isomers, but the control methods and limits of its configurational isomers are not recorded in the Chinese Pharmacopoeia 2015, vol, and Japanese Pharmacopoeia 2016, vol. For this reason, the established method did not take the configurational isomers into account.

The use of HPLC for the analyte detection, usually requires a previous treatment of the samples causing the loss of information. In contrast, the MD technique allows simultaneous collection of samples from multiple sites without the need of pretreating it because the dialysis membrane excludes proteins from the aqueous phase. So, the samples obtained by MD sampling technique could be directly analyzed using the LC-MS/MS method. Since an IS is important to assess the analyte content in biological samples, some compounds were made as a comparison and screen at the beginning of the experiment, and then NCG, an analog of NAG, was selected to be the optimal IS due to its stable property, well resolution, and reduction of interference in the dialysate matrix (Stuehr, 2004; Tang et al., 2014).

Because MD sampling is a non-equilibrium process, the concentration of drug in the dialysate is some fraction of the actual extracellular protein-unbound drug concentration (Sun et al., 2001). For quantitative measurement, MD probes thus need to be calibrated *in vivo* to obtain a factor for the relative drug transfer across the membrane, i.e. an *in vivo* recovery value (Brunner et al., 2000). The MD probe recovery could be influenced by the following experimental elements including the length, diameter and materials of the membrane, effective surface area, probe geometry, the diffusion coefficient of the analyte, the component of perfusion solution, the flow rate for perfusion, time, temperature, and the property of the substance. For MD *in vivo*, the tissue environment has additional effects on the recovery (Sun et al., 2001; Hou et al., 2016). In the present study, MD was successfully conducted for the measurement of NAG in blood as well as brain tissue. In the preliminary *in vitro* study, we found that RR was equal to RL at different concentrations and was independent of concentration. The *in vivo* recovery is consistent with the *in vitro* recovery of NAG at the same flow rate. Based on these findings, *in vivo* NAG experiment was considered feasible (Deng et al., 2018).

The BBB is a highly dynamic barrier to maintain the homeostasis of brain parenchymal microenvironment because of its tight junctions, low pinocytotic activity, efficient transporters, and its metabolic properties (Gustafsson et al., 2017). The drug molecule entering the CNS is restricted due to its the exceptional ability which is also the major hindrance in CNS drug development. Meanwhile, drug transport into the brain is limited by the BBB and related to physic-chemical factors of the drug including lipophilicity, ionization, and pH in relation to membrane property. The concentration-time profile and the concentration level in brain in turn determine the pharmacodynamic effect over time (Hammarlund-Udenaes et al., 1997). The relationship between brain and blood levels of NAG, yields higher the brain/blood ratio in NAG-H and GHI groups than NAG-L, NAG-M groups (**Table 8**), indicating NAG has appreciable BBB penetrability. It is vital to grasp the time course of the neuroprotective activity of NAG by detecting the drug concentration, both in rat blood and brain. Moreover, the pharmacokinetic characteristics of NAG were helpful to determine the administration regimen to ensure better efficacy. Our research indicated that NAG could throughout the BBB and T_1/2_ values of all groups in blood were lower than that in brain, suggesting that NAG was eliminated faster in the blood than that in the brain.

Bergana et al. identified that the decomposition compounds of NAG at low pH were glutamine, Glu, pyroglutamic acid, and N-acetylglutamic acid (Bergana et al., 2000). Previous researches show that NAG can penetrate the BBB and decompose into Glu and GABA which play vital roles in maintaining the balance of brain function. Glu is the major excitatory amino acid neurotransmitter in brain. More than 50% neurotransmitter of synapses is Glu. Almost the excitatory neurons are glutamatergic neurons. It is crucial that Glu maintains the normal excitability of neurons, but excess excitatory Glu causes the destruction of caudate/putamen dopamine nerve ending and neurodegeneration of the thalamus, parietal cortex, and limbic system. Superabundant Glu also produces excitotoxic effects on ischemic neurons (Lee and Kesner, 2002; Gabriele et al., 2012).

As the most important inhibitory neurotransmitter in the CNS, GABA hyperpolarizes the nerve membrane potential to inhibit the release of Glu energy transmitters, blocks the proliferation and transmission of excitement, thereby inhibits the excitatory neurotoxicity of excess Glu to maintain the balance of brain function (Costa et al., 2004). The extracellular balance of Glu and GABA is especially important in pathological processes including epilepsy, excitotoxic degeneration, and other neurodegenerative disorders. So as a brain function improving agent, NAG decomposes the excitatory Glu and the inhibitory GABA to sustain homeostatic mechanisms of brain. This study investigated the unbound concentration of NAG, together with the metabolites Glu and GABA after drug administration. In the medium, high doses of NAG and GHI groups, the T_1/2_ values in brain microdialysate were significantly lower than those of the corresponding groups in blood. The blood-brain permeability of GHI was much lower than that of other drug groups. The results indicate that GHI accelerates the elimination of Glu *in vivo*, inhibits Glu from crossing the BBB. The blood-brain permeability of GABA remarkably increased compared with Glu in drug groups indicating that NAG and GHI increase the content of GABA in the brain to counteract excessive Glu and maintain the balance of brain function.

The concentration of aceglutamide in GHI is 30 mg/ml, and the commonly used clinical dose is 10~20 ml through intravenous injection. In addition, according to the body surface area normalization method which was recommended by the Food and Drug Administration (Reagan-Shaw et al., 2008), the dose of NAG used in our experiment was equivalent to an approximate dose of 12 mg/kg in humans, which is considered the usual dose of clinical practice.

## Conclusion

In this paper, a LC-MS/MS method combined with MD technique was established to quantify the NAG and its metabolites Glu and GABA in rat blood and brain tissues. The screening of mobile phase was performed at first and acetonitrile was chosen as the organic phase. Under the optimized LC conditions, the analytes and the interferences in the blank matrix were well separated. Then, the validity of the method was assessed by methodology investigation including the specificity, linearity, precision and accuracy, ME and sensitivity, stability, and recovery. Then the blood pharmacokinetics and brain distribution kinetics of NAG, Glu, and GABA were studied. The concentration-time profiles and pharmacokinetic data for NAG, Glu, and GABA in blood and brain were detected to estimate the BBB penetration of NAG, Glu, and GABA. The results indicate that the established method can be successfully employed to study the pharmacokinetic of NAG and its related metabolites in rat. Current data obtained from rat suggest that NAG has easy and dose-dependently access to the BBB and exhibits a medium retention time. Accordingly, a pivotal pharmacological foundation for the effective clinical use of NAG and the establishment of a theoretical basis for GHI to treat cerebrovascular diseases are provided by our study.

## Data Availability Statement

All datasets generated for this study are included in the article/supplementary material.

## Ethics Statement

The animal study was reviewed and approved by the institutional animal care and use committee of Zhejiang Chinese Medical University.

## Author Contributions

YH, SX and LD conceived and designed the study and SX, LD accomplished the experiment. LY, HZ tested and analyzed the data. SX and LD wrote this manuscript. YH, LY, and HW coordinated the research and provided the technical assistance. All authors reviewed the results and approved the final version of the manuscript.

## Funding

Natural Science Foundation of Zhejiang Province (Nos. LZ18H270001, LQ19H280004); National Natural Science Foundation of China (Nos. 81873226, 81630105); The Traditional Chinese Medicine Science and Technology Plan of Zhejiang Province (No. 2018ZQ013); Zhejiang Provincial Science and Technology Innovation Leading Talent Project of “Ten Thousand Talents Plan” (2019).

## Conflict of Interest

The authors declare that the research was conducted in the absence of any commercial or financial relationships that could be construed as a potential conflict of interest.

## Abbreviations

NAG, acetylglutamine; Glu, glutamic acid; GABA, γ-aminobutyric acid; MD, microdialysis; GHI, Guhong injection; NCG, N-carbamyl-L-glutamic; LC-MS/MS, liquid chromatography-tandem mass spectrometric; CNS, central nervous system; BBB, blood-brain barrier; MRM, multiple reactions monitoring; QCs, quality control samples; RSD, relative standard deviation; RE, relative error; RR *_in vivo_*, *in vivo* relative recovery; RR *_in vitro_*, *in vitro* recovery; RL *_in vitro_*, *in vitro* loss; RL *_in vivo_*, *in vivo* loss.
